# SARS-CoV-2 Reinfections and Long COVID in the Post-Omicron Phase of the Pandemic

**DOI:** 10.3390/ijms241612962

**Published:** 2023-08-19

**Authors:** Fotini Boufidou, Snežana Medić, Vicky Lampropoulou, Nikolaos Siafakas, Athanasios Tsakris, Cleo Anastassopoulou

**Affiliations:** 1 Neurochemistry and Biological Markers Unit, 1st Department of Neurology, Eginition Hospital, School of Medicine, National and Kapodistrian University of Athens, 11528 Athens, Greece; fboufidou@med.uoa.gr; 2 Department of Epidemiology, Faculty of Medicine, University of Novi Sad, 21000 Novi Sad, Serbia; snezana.medic@mf.uns.ac.rs; 3 Center for Disease Control and Prevention, Institute of Public Health of Vojvodina, 21000 Novi Sad, Serbia; 4 Department of Microbiology, Medical School, National and Kapodistrian University of Athens, 11527 Athens, Greece; vlampro@med.uoa.gr (V.L.); atsakris@med.uoa.gr (A.T.); 5 Department of Clinical Microbiology, Attikon General Hospital, Medical School, National and Kapodistrian University of Athens, 12462 Athens, Greece; nsiaf@med.uoa.gr

**Keywords:** COVID-19, long COVID, long-haul COVID, late sequelae, post-acute sequelae of SARS-CoV-2 infection (PASC), ME/CFS, reinfection severity, vaccination benefits, pediatric long COVID

## Abstract

We are reviewing the current state of knowledge on the virological and immunological correlates of long COVID, focusing on recent evidence for the possible association between the increasing number of SARS-CoV-2 reinfections and the parallel pandemic of long COVID. The severity of reinfections largely depends on the severity of the initial episode; in turn, this is determined both by a combination of genetic factors, particularly related to the innate immune response, and by the pathogenicity of the specific variant, especially its ability to infect and induce syncytia formation at the lower respiratory tract. The cumulative risk of long COVID as well as of various cardiac, pulmonary, or neurological complications increases proportionally to the number of SARS-CoV-2 infections, primarily in the elderly. Therefore, the number of long COVID cases is expected to remain high in the future. Reinfections apparently increase the likelihood of long COVID, but less so if they are mild or asymptomatic as in children and adolescents. Strategies to prevent SARS-CoV-2 reinfections are urgently needed, primarily among older adults who have a higher burden of comorbidities. Follow-up studies using an established case definition and precise diagnostic criteria of long COVID in people with or without reinfection may further elucidate the contribution of SARS-CoV-2 reinfections to the long COVID burden. Although accumulating evidence supports vaccination, both before and after the SARS-CoV-2 infection, as a preventive strategy to reduce the risk of long COVID, more robust comparative observational studies, including randomized trials, are needed to provide conclusive evidence of the effectiveness of vaccination in preventing or mitigating long COVID in all age groups. Thankfully, answers not only on the prevention, but also on treatment options and rates of recovery from long COVID are gradually starting to emerge.

## 1. Introduction 

The set of different symptoms that persist after infection with acute respiratory syndrome coronavirus 2 (SARS-CoV-2) is referred to in the literature under different names. The umbrella term “post-COVID conditions” (PCC) is used by the Centers for Disease Control and Prevention (CDC) for the wide range of health consequences that can present after four or more weeks, even after mild or asymptomatic infection with SARS-CoV-2 [1]. The post-acute sequelae of SARS-CoV-2 infection (PASC) that are also commonly referred to as “long COVID” or “long-haul COVID” may comprise a wide spectrum often of severe symptoms, ranging from fatigue and shortness of breath to headaches and difficulty in concentrating (the so-called “brain fog”). An overview of long COVID has been recently published by Davis et al. [2].

Attempts have been made to develop a definition for long COVID and classify the associated clinical phenotypes [3,4]. However, the heterogeneity that characterizes its symptom trajectories and the overlap with other medical conditions, in conjunction with the absence of a diagnostic biomarker or imaging, render this task particularly challenging. Consequently, it is not surprising that it was not until October 2021 that a code for long COVID was added in the International Classification of Diseases 10th Edition, with concerns remaining about its sensitivity and specificity. The World Health Organization (WHO) uses a rather broad definition, “symptoms arising or continuing within three months of infection with SARS-CoV-2 and lasting at least two months with no other explanation”, that is, nonetheless, disputed [5].

**Figure 1:**
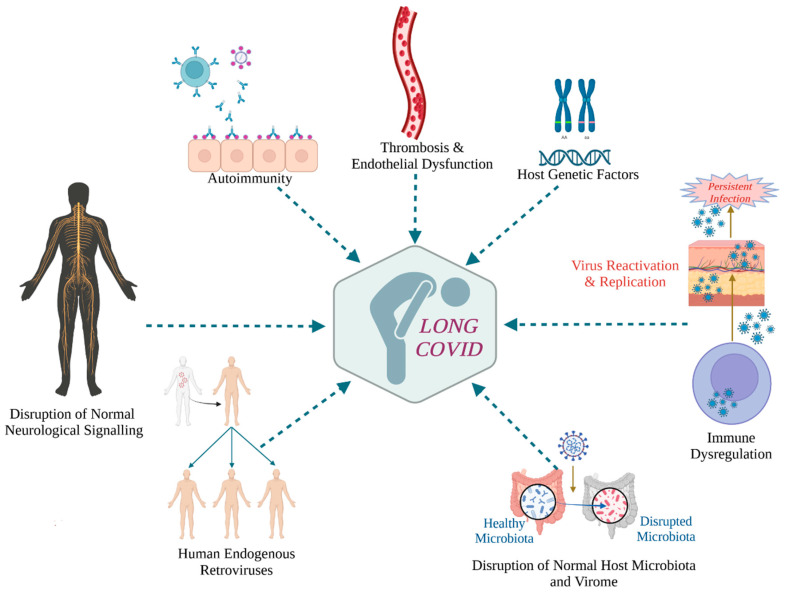
Proposed underlying pathogenetic mechanisms of long COVID that include both host and viral factors. Created with BioRender.com (accessed on 3 April 2023).

Undisputed is the public health significance of long COVID given the large number of individuals who suffer from long COVID that amounts to at least 65 million globally, based on conservative estimates [2]. Thus, even though the emergency phase of the COVID-19 pandemic is over, new SARS-CoV-2 variants continue to emerge, leading to new infections and reinfections, possibly but not necessarily of reduced severity, as well as to post-acute phase complications that may be severe and persist for months or years [2]. Thus, it is important to understand the pathophysiological mechanisms of long COVID so as to find effective therapeutic protocols for the many patients who suffer from post-infection sequelae. Older age, female sex, obesity, smoking, and certain comorbidities, such as diabetes, as well as having had a severe episode of COVID-19, are risk factors for long COVID [2]. Several underlying pathogenetic mechanisms of long COVID have been proposed [2,6,7], involving both viral and host factors, as outlined in Figure 1.

In this narrative review of the literature, the first one of its kind to the best of our knowledge, we summarize the current state of knowledge on the virological and immunological correlates of long COVID. Although long COVID has caused global concern, currently there are very limited published studies investigating the long-term consequences of SARS-CoV-2 reinfection. Therefore, we also present recent evidence on the possible association between the increasingly common SARS-CoV-2 reinfections and the parallel pandemic of long COVID that is unfolding in the post-Omicron era. Finally, we critically discuss remaining challenges and unknowns regarding this complex condition with respect to the potential preventive benefit of vaccination and children and adolescents as a population group of special interest that may also suffer from long COVID. Although SARS-CoV-2 reinfections tend to be milder than primary infections, the risk of developing long COVID remains.

## 2. Literature Search Methodology

We searched PubMed/MEDLINE for all English-language original articles or reviews reporting on potential associations between SARS-CoV-2 reinfections and long COVID, up to 11 August 2023 (during the revision of the manuscript). Articles on preprint servers (i.e., BioRxiv and MedRxiv) were included in our search. The search was performed using all combinations of terms related to SARS-CoV-2 and its circulating variants (e.g., “Omicron” or “Delta”) on one hand, and the different names used for long COVID (e.g., “PCC”, “PASC”, “long-haul COVID”, as well as the terms “vaccines” or “vaccination” and “children and adolescents”, “pediatric long COVID”), on the other. All study types and countries of origin, irrespective of the size of patients’ cohorts or whether positive or negative results were reported, were included in our study.

## 3. Virological Correlates of Long COVID

### 3.1. A Common Mechanism of Unexplained Post-Acute Infection Syndromes?

The Mechanistic Pathways task force of the “RECOVER” (Researching COVID to Enhance Recovery) consortium, a National Institutes of Health (NIH)-sponsored initiative that seeks to understand the basis of long COVID in a large cohort, has recently published a detailed review article on viral persistence and reactivation as potential underlying mechanisms of long COVID [8]. Viruses may persist in forms that are not well understood following the potentially slow resolution of viral RNA or antigen from the respiratory epithelia or other tissue sites. A persistent reservoir or non-infectious remnants in deep tissues could generate pathogen-associated molecular patterns (PAMPs), such as viral RNA, which might engage various host pattern recognition receptors (PRRs) to trigger innate immune activation and potentially also T and B cells [9]. The result can be chronic stimulation of these lymphocytes if the effector functions of T cells and antibodies are insufficient to clear the pathogen, which could lead to inflammatory conditions. Inflammatory responses to SARS-CoV-2 can co-exist with re-activation of other latent viruses. Reactivated viruses may include herpesviruses, such as Epstein–Barr virus (EBV), a virus that has been suggested to be associated with chronic fatigue or other symptoms that resemble long COVID symptoms [8]. The many similarities between long COVID and chronic illnesses triggered post infection with other pathogens in a minority of patients who present with symptoms resembling myalgic encephalomyelitis/chronic fatigue syndrome (ME/CFS), suggest the potential involvement of a common etiopathogenesis [9].

### 3.2. Viral Persistence and Long COVID

Persistence of SARS-CoV-2-infected cells or components of the virus, such as the spike protein, has been described [10,11,12]. The human angiotensin-converting enzyme 2 (ACE2), which serves as a receptor for viral entry into the host cell along with cellular serine proteases that prime the spike protein for fusion with the cellular membrane, are expressed not only along the human respiratory system, but also in a wide range of cell types that include the brain endothelium, vascular smooth muscle cells, gastrointestinal epithelial cells, renal proximal tubules, esophageal keratinocytes and pancreatic β-cells [13,14,15]. Therefore, if prolonged sustainment of the multi-system tissue tropism of SARS-CoV-2 could be proven in long COVID patients, significant evidence for the role of viral persistence would be obtained.

So far, frequent detection of spike proteins in plasma samples of long COVID patients at any time point between 2–12 months post-infection has been reported [16]. Moreover, prolonged viral shedding through the gastrointestinal tract has been shown by molecular detection of SARS-CoV-2 RNA in stool samples for extended periods, providing evidence of virus persistence [17]. The presence of viral genetic material and proteins has also been recorded in the cardiovascular system, reproductive system, cerebrospinal fluid (CSF), brain, muscles, eyes, lymph nodes, appendix, breast tissue, liver, lung, and urine [18,19,20,21,22,23,24].

However, the mechanisms of viral persistence in individuals remain elusive. Uncovering these mechanisms would provide the rationale for guiding treatment [8]. For instance, if the viral replication machinery is implicated in persistence, then using antivirals would make sense. If that is not the case, it would probably be beneficial to use vaccines or other immunomodulators to target persisting virus. Viral persistence may be the result of incomplete immunity, where viral antigen and RNA slip through “holes” of the immune response. Furthermore, evidence has been provided in the literature that pathology is caused from persistent inflammatory responses to persistent antigen [8].

### 3.3. Viral Reactivation *and Long COVID*

Suppression or dysregulation of the host immune response by SARS-CoV-2 during the acute phase of infection is a complex process that may allow viruses already present in a latent state to reactivate, and possibly to infect new tissues and organs within the host, thereby eliciting new chronic symptoms [7]. For instance, a study of the human virome in healthy individuals [25] identified the presence of many viral species in at least one tissue of healthy individuals, including the brain, heart, pituitary gland, lung, kidney, nervous system, and many others. The viruses identified included the known herpesviruses EBV, HSV, CMV, VZV, and HHV-6, HHV-7, as well as viruses from other families such as HCV, HPV, adenoviruses, and several respiratory viruses. SARS-CoV-2 can disrupt this dynamic equilibrium of asymptomatic carriage and lead to the reactivation of other co-infecting viruses [26], which could contribute to the persistent sequelae of long COVID [2]. In addition, some human endogenous retroviruses (HERVs) have been associated with more severe acute SARS-CoV-2 infection [27,28].

## 4. Immunological Correlates of Long COVID

Defining the immunological correlates of the long-term symptoms that persist or manifest after COVID-19 is under investigation. Different hypotheses have been proposed, including persistent SARS-CoV-2 proteins and/or RNA driving chronic inflammation, triggering of autoimmunity after acute SARS-CoV-2 infection, unrepaired tissue damage, and microbiome/virome dysbiosis [7]. Aberrant innate and adaptive immune responses have been implicated in long COVID. The findings reviewed here are from studies of long COVID patients who had been infected with the original Wuhan SARS-CoV-2 strain and had not been vaccinated at sampling points or their vaccination status was accounted for in the reported analyses [29,30].

### 4.1. Innate Immune Cell Dysregulation

Innate immune cell dysregulation has been described in studies comparing patients with long COVID with uninfected controls and infected patients without long COVID at 13–15 months post mild infection [10,29]. Long COVID patients had higher numbers of peripheral non classical monocytes—which can mediate vascular homeostasis and anti-viral immune responses—and decreased abundance of the conventional dendritic cell subset cDC1 that is typically involved in antigen cross-presentation to CD8^+^ T cells and priming of anti-viral T_H_1 cell responses [29]. Persistent innate immune responses in long COVID are also supported by findings of increased plasma levels of cytokines, specifically IL-6, TNF, and IL-1β [30,31,32,33], likely produced by monocytes and/or dendritic cells [30,32]. Longitudinal analyses of sera proteomes revealed in addition elevated IFN levels of type II (IFNγ) and III (IFNλ1) in long COVID patients that remained throughout the acute and post-acute disease phases (>60 days) [32], compared to symptom-free convalescent individuals. In another long COVID cohort, increased concentrations of circulating IFNλ1 and IFNβ (type I) remained higher up to eight months after infection, whereas in the fully recovered these responses declined over time [34].

Collectively, in view of these reported differences, establishing an objective quantification and a universal concentration threshold of individual cytokines that can be of diagnostic or predictive value for bulk or individual long COVID symptoms would be clinically useful, although conceivably challenging. Machine learning approaches have suggested minimal sets of analytes that could serve as biomarkers with relatively high accuracy, for example increased serum IL-8 and galectin-1 together with low cortisol levels [29], or combined increases in serum IFNβ, PTX3, IFNλ2/3, and IFNγ [34], although these remain to be confirmed in larger cohorts.

#### CCL11/Eotaxin1 and Neurological Long COVID Symptoms

A recent study found increased plasma levels of CCL11/eotaxin1 in a subset of a long COVID cohort with prominent cognitive symptoms—referred to as “brain fog”—compared to long haulers that lacked these symptoms [35]. CCL11, well-known as an eosinophil chemoattractant, has been implicated in cognitive and memory deficits related to aging and certain psychiatric and neurodegenerative disorders, and has been shown to impair neurogenesis in mice [36,37,38]. Its presence in larger long COVID cohorts with similar neurological symptoms, mechanism of action, and causality to specific neurological long COVID symptoms await further investigation.

### 4.2. Dysregulated Adaptive T Cell Responses

Dysregulated adaptive T cell responses have been reported across different long COVID cohorts. Cross-sectional studies of long COVID patients who had mild infection have found decreases in central memory [29] and effector memory CD4^+^ T cells [39], plus higher frequencies of exhausted CD4^+^ and CD8^+^ T cells [29], as well as increased expression of PD-1 on central memory T cells [39], an inhibitory molecule associated with T cell exhaustion in chronic viral infection and cancer. Long haulers also had increased frequencies of T cell subsets that co-produced IL-4 and IL-6 after polyclonal stimulation [29], further implicating aberrant immunological conditioning in long COVID. Other studies reported insufficient anti-viral T cell responses in long COVID patients, including lower abundance of nucleocapsid-specific CD8^+^ T cells expressing CD107a (a degranulation marker) at 4 months post infection [40] and reduced or absent SARS-CoV-2-specific CD4^+^ and CD8^+^ T cells in severe long COVID [32]. Longitudinal analyses showed a faster decline in nucleocapsid (N)-specific IFNγ-producing CD8^+^ T cells in patients with long COVID compared to the fully recovered, while the N-specific IFNγ^+^CD4^+^ T cell responses were similar [40].

Further immunological characterizations have indicated distinct correlations of antigen-specific T cell responses with different long COVID symptoms. Prolonged lung dysfunction correlated with increased TNFα^+^ and IFNγ^+^ SARS-CoV-2-specific T cells and systemic IL-6 production regardless of patients’ hospitalization status during acute infection [33]. Persistent manifestation of neurological long COVID correlated with enhanced CD8^+^ T cell responses to membrane (N) but not spike (S) viral proteins compared to complete convalescence at 6 months post infection [41], whereas post-acute expansion of new SARS-CoV-2-specific CD4^+^ and cytotoxic CD8^+^ T cell clones was associated with gastrointestinal (GI) long COVID [42]. Patients experiencing GI long COVID also had persistent CMV-specific CD8^+^ T cells—in the absence of CMV viraemia—expressing a more pronounced cytotoxicity transcriptomic signature compared to CMV-specific CD8^+^ T cells from healthy SARS-CoV-2-unexposed individuals [42], implicating bystander T cell activation in long COVID, as previously noted in mild acute COVID-19 patients [43].

### 4.3. Aberrant B Cell and Antibody Responses

Differing magnitude and distinct patterns of SARS-CoV-2-specific and non-specific B cell and antibody responses have been described in long COVID patients and/or associated with risk of long COVID development. A multidimensional comparison of long COVID with uninfected and fully recovered individuals revealed increased frequencies of activated B cells, double-negative B cells, and higher levels of anti-S and S1 IgG with enhanced and unique binding targets in the long COVID group [29]. These elevated humoral responses positively associated with exhausted T cells indicating persistent anti-viral immune responses in long COVID [29]. In the same study, long haulers also exhibited increased levels of IgG antibodies against EBV antigens which is indicative of recent reactivation of latent EBV [29], although a corresponding increase in CMV-specific T cells that normally control EBV reactivation was not examined [29].

Patients with neurological long COVID had elevated IgG and T follicular helper cells specific for N protein, but not spike RBD, compared to convalescent controls at 6 months after mild acute infection [41], and slightly increased anti-N IgG titers significantly correlated with neurological long COVID also after severe COVID-19 [42]. Other studies showed that long COVID at 2 months post infection negatively correlates with the magnitude of RBD-specific memory B cell and anti-N IgG responses [32], and that weaker anti-S1 IgG production during mild acute infection could be predictive of long COVID symptoms [44,45]. Yet, in separate cohorts, SARS-CoV-2 neutralizing antibody levels were similar between long COVID and fully recovered patients at 8 months after mild acute infection [40,46] and, as expected, anti-Spike antibodies correlated with anti-Spike CD4^+^ T cell responses [40].

### 4.4. Autoreactive Antibodies

Increased and de novo production of autoreactive antibodies against intracellular or extracellular self-antigens and/or antibodies that cross-react with SARS-CoV-2 and self-proteins have been described in mild [47] and moderate/severe [48,49] COVID-19 patients (compared to uninfected controls). Collectively, diverse self-reactivities with functional potential have been identified that target: (i) tissues and organs, including connective tissue, extracellular matrix, vascular endothelium, skin, lung, GI tract, central nervous system (CNS); (ii) extracellular antigens, signaling receptors and immunomodulatory proteins, such as chemokines, cytokines, surface proteins; (iii) intracellular proteins, including nuclear antigens (e.g., Ro, La) and dsDNA, cardiolipin, myeloperoxidase, and others. It has been hypothesized that such autoantibodies persist post recovery driving or contributing to the manifestation of prolonged post-acute symptoms. Consequently, the presence and potential association of autoantibodies with the development of long COVID has been examined in several cohorts.

Autoantibodies to ACE2 have been detected in convalescent COVID-19 patients and correlated with the production of RBD IgG antibodies [50]. Functional autoantibodies to 2–7 G-protein Coupled Receptors (GPCR) including muscarinic M2 receptors and angiotensin II AT1 receptors were found in patients with persistent neurological and cardiovascular long COVID [51]. In addition to autoimmunity, GPCR autoantibodies have been associated with cardiovascular, pulmonary, and CNS diseases [52]; therefore, they could promote related long COVID symptoms. A multiomic longitudinal analysis of mostly hospitalized COVID-19 patients followed up to 2–3 months post infection identified IgG autoantibodies against intracellular antigens at initial diagnosis as a PASC-anticipating factor, along with type 2 diabetes, SARS-CoV-2, and EBV viremia [42]. It also pointed at an association with atypical memory B cells, exhibiting lower levels of somatic hypermutation and enhanced BCR and IFN signaling in long COVID, sharing pathogenic mechanisms with systemic lupus erythematosus. In the same study, autoantibodies anti-correlated with protective anti-SARS-CoV-2 antibodies, and certain self-reactivities were associated with different PASC. These anti-correlations were different and weaker in a validation long COVID cohort of participants who had mostly mild acute infection and lower anti-spike humoral responses [42].

In other studies, autoantibodies to intracellular antigens and rheumatoid factor (RF) or to extracellular/secreted proteins were not found to associate with long COVID symptoms at 8 or 14 months after mild acute COVID-19 [29,30]. These discrepancies could be attributed to differences in sampling points, as autoantibody responses may wane after several months. Alternatively, they could raise the notion that a pathogenic role for autoantibodies is more prominent in long COVID that develops after more severe acute infection at least with the original Wuhan strain or in patients with comorbidities that predispose to more severe disease, including type 2 diabetes, cardiovascular diseases, and obesity. Nonetheless, the findings of private antibody autoreactivities during and after mild infection [29,47] may point to the need to further refine the long COVID cohorts. Lastly, in addition to autoantibodies, it might be worth investigating the activation of autoreactive T cells, which can be triggered during infections via different mechanisms [53], and which could also contribute to tissue damage and long COVID in some individuals.

## 5. SARS-CoV-2 Reinfections and Long COVID

### 5.1. Do Repeated Infections with SARS-CoV-2 Increase the Likelihood of Long COVID?

Studies by our group and others showed that SARS-CoV-2 reinfections were rare before the advent of Omicron and increased significantly thereafter [54]. Obtaining a precise estimate of the frequency and severity of SARS-CoV-2 reinfections is not feasible due to differences in the case definition of reinfection, testing protocols and criteria, study methodology, and availability of tests during the pandemic. Such differences do not allow for direct international comparisons of reinfection rates. Most reinfections tend to be mild, and associated hospitalizations and deaths are very uncommon [54]. However, even mild or asymptomatic infections and reinfections can lead to long COVID.

The potential impact of reinfections on increasing the likelihood of long COVID, or on exacerbating pre-existing long COVID, are key questions as the pandemic potentially moves into an endemic phase. Long COVID patients with low levels of protective antibodies and increased levels of autoantibodies are possibly more prone to SARS-CoV-2 reinfections [2]. The cumulative risk of long COVID was found to increase in proportion to the number of SARS-CoV-2 infections, when compared with no reinfection, primarily in older age [55]. Furthermore, compared with those infected once, patients who were reinfected were more prone not only to long COVID, but also to various complications, including potential cardiac, pulmonary, or neurological problems.

### 5.2. Long COVID Following Reinfection with SARS-CoV-2 Variants Pre- and Post-Omicron

The number of long COVID cases is expected to increase in the future as SARS-CoV-2 reinfections become more frequent in the current post-omicron period, resulting in a parallel long COVID pandemic. Some studies have shown an increased risk of long COVID after SARS-CoV-2 reinfection even in vaccinated individuals [2]. Both symptomatic and asymptomatic SARS-CoV-2 reinfections may result in long COVID, but the risk of developing long COVID symptoms in the asymptomatic group was significantly lower [56]. The notification rate of long COVID has been increasing for reinfections caused by more recent SARS-CoV-2 subvariants of the Omicron family [57,58]. Omicron’s high variability (particularly for BA.4 and BA.5 subvariants) and higher risk of reinfection compared to Beta (B.1.351) and Delta (B.1.617.2) variants raise concerns about immunity escape, regardless of its origin [59].

Unlike previous SARS-CoV-2 variants where the incidence of long COVID following reinfection was lower than after the initial infection, a higher incidence of long COVID after a first Omicron BA reinfection was observed than after an initial Omicron BA infection. The incidence rate of long COVID following the first episode was highest in the Delta variant period and lowest in the Omicron BA period [57]. The detection of Delta, possibly the most pathogenic SARS-CoV-2 variant characterized thus far, was accompanied by a surge in COVID-19-associated mucormycosis cases, mainly affecting patients with poorly controlled diabetes mellitus particularly in India, Iran, and Egypt, adding significant burden to COVID-19 [58,60]. Recent studies indicated a reduction in the odds of long COVID with Omicron compared to the Delta variant, depending on age and time since vaccination [58,60]. However, the absolute number of cases of long COVID is incomparably higher as a consequence of the larger magnitude of the pandemic curve caused by the Omicron SARS-CoV-2 family with its many subvariants that led to the highest percentage of reinfections since the beginning of the pandemic.

Some specific host and viral risk factors potentially associated with increased chances of long COVID after SARS-CoV-2 reinfections [58,59,60,61,62,63] are summarized in Table 1.

### 5.3. Future Directions on the Study of the Impact of Reinfections on Long COVID

A possible link between SARS-CoV-2 reinfections and the consequent higher likelihood of long COVID becomes crucial as the COVID-19 pandemic is now waning amid widespread relaxation of social distancing and other restrictions, causing many people to become infected multiple times. However, the number of studies providing evidence of long COVID following SARS-CoV-2 reinfection is limited. The study by Bowe et al. indicated that reinfection further increases the risk of long COVID sequelae in the acute and post-acute phase, necessitating strategies to prevent SARS-CoV-2 reinfection, primarily in the older-age population with the higher burden of comorbidities [55]. Natural immunity after primary infection with the pre-omicron SARS-CoV-2 variants provides high and long-lasting protection against reinfection even after 40 weeks [61]. However, the protection is significantly lower for Omicron BA.1 and decreases faster over time compared to previous variants, resulting in a substantial increase of reinfections. Although available data suggest that infection with Omicron variants leads to a lower risk of developing long COVID compared to previous historical SARS-CoV-2 variants, more recent data show higher odds of long COVID following Omicron BA reinfections, predominantly in older age [55].

The small number of studies as well as the short follow-up time limits the generalizability of these findings [58]. Immunity acquired by previous infection should be weighed together with protection from vaccination when creating a vaccination policy to prevent long COVID in the future. Further follow-up studies using the established case definition and precise diagnostic criteria for long COVID in people with or without reinfection, in both vaccinated and unvaccinated cohorts, may help to better understand the contribution of SARS-CoV-2 reinfections to long COVID burden. Moreover, prospective cohort studies with long observational periods for patients who experienced reinfections during different pandemic waves would be helpful to follow the evolution of long COVID [62].

### 5.4. Strategies to Prevent SARS-CoV-2 Reinfections

Reinfection has been shown to further increase risks of all-cause mortality and adverse health outcomes in both the acute and post-acute phases of reinfection; therefore, strategies to reduce the risk of reinfection are urgently needed [55]. Non-pharmaceutical interventions towards this aim would include avoiding SARS-CoV-2 infection and reinfection, mostly by adopting a number of basic measures against respiratory viral infections, such as wearing facial masks that fit well around the nose and mouth and applying hand hygiene and social distancing [1,54,55]. Proper ventilation of indoor spaces, especially of schools, offices, and commercial buildings, but also of homes, could also help. In addition, pharmaceutical interventions could include more durable vaccines that would cover a broad array of variants and reduce transmission, thus also reducing the risk of infection and reinfection as well as both acute and long-term adverse health consequences [8,55].

## 6. Vaccination and Long COVID

### 6.1. Does Vaccination against COVID-19 Protect from Long COVID?

The swift development of several highly effective vaccines against severe COVID-19, just over a year after the emergence of SARS-CoV-2, was an amazing scientific accomplishment [2,62]. Advances in nanotechnology allowed for the use of novel vaccine platforms, as in the case of mRNA-based vaccines that include Pfizer-BioNTech’s BNT162b2 (Comirnaty, Tozinameran) and Moderna’s mRNA-1273 (Spikevax, CX-024414) [2,62]. The primary advantage of nanovaccines lies in the usage of organic nanoparticles (NPs) in their formulations for the protection and delivery of the active substance, which can be a recombinant spike protein or a nucleic acid (e.g., an mRNA molecule that encodes the viral antigen, the SARS-CoV-2 whole-spike protein or its subunits) [2]. Vaccines against severe COVID-19 have helped to mitigate the devastating effects of the pandemic [2].

Vaccination against COVID-19 may also limit the long COVID burden by reducing the odds of SARS-CoV-2 infection, limiting disease severity upon infection, and perhaps by improving the course of long COVID [63]. Despite the large number of studies on this subject, results are not always directly comparable due to differences in research methodology, types of vaccines, number of doses, and time of onset of symptoms [64]. Vaccine effectiveness, the number of doses, and the elapsed time since the last dose and exposure, as well as the variant of SARS-CoV-2, can affect the occurrence of long COVID [2]. Although some studies have not proven that prior vaccination protects against long COVID [65], others have provided evidence of a reduced risk of long COVID between 15% and 41% [66,67].

Overall, preliminary evidence (grade III) suggests that vaccination before SARS-CoV-2 infection is associated with lower risks and odds of long COVID and that two vaccine doses are more effective than one dose [68,69]. The prevalence of long COVID is estimated at 10–12% of vaccinated patients [2]. Although the majority of vaccinees reported improvement in long COVID symptoms without difference in relation to the type of vaccine received [70], other studies have shown conflicting results on the impact of vaccination on pre-existing long COVID [2,68,71].

### 6.2. The Benefits of Vaccination with Respect to Long COVID

A reduced risk of long COVID in people vaccinated with two vaccine doses before or after COVID-19 compared to unvaccinated individuals is supported by several studies [60,69,72,73,74]. A retrospective national cohort study from Israel showed that the risk of long-term dyspnea was lower in vaccinated with breakthrough infection compared with the unvaccinated cohort, while the risks of all other outcomes were similar [74]. A lower risk of chronic fatigue and pulmonary disorder was associated with a two-dose vaccination series compared to no vaccination [75]. Based on the available evidence, the complete vaccination series (in the initial scheme) is likely to reduce the incidence, severity, and duration of long COVID, and to provide additional protection over a single dose of vaccine [64,70]. Long COVID is less likely in those infected with newer viral variants and in those who have received three doses of a COVID-19 vaccine (level of evidence = 1 b) [76].

A systematic review with meta-analysis that explored the effect of COVID-19 vaccination on long COVID showed that the vaccinated group had a 29% lower risk of developing long COVID compared to the unvaccinated group; moreover, the risk of cognitive dysfunction, kidney disease, myalgia, and sleep disorders was also reduced in the vaccinated [69]. Vaccination prior to COVID-19 onset has a moderate but consistent protective association with long COVID even in the case of breakthrough infections [77]. In a study of patients discharged from hospital, non- or incomplete vaccination, female sex, and admission to intensive care during hospitalization were associated with ≥1 symptoms 90 days after hospital discharge, suggesting that even in patients with severe COVID-19, vaccination reduces the likelihood of long COVID [78].

The greatest benefit of vaccination appears to be in reducing the odds of blood clotting and pulmonary complications, but there was no difference between the vaccinated and the unvaccinated in a range of other conditions, including neurological, kidney, and gastrointestinal problems [66]. Although more data need to be collected, early evidence suggests that vaccination is more effective in preventing long COVID in the elderly [64]. Some studies show improvement in pre-existing long COVID after vaccination. An observational cohort study of UK adults has shown that vaccination may reduce the long COVID health burden with a sustained improvement after a second dose at least a few months after vaccination, yet longer follow-up is needed [71].

In addition, SARS-CoV-2 infection after a two-dose vaccine series was associated with a reduced risk of developing long COVID at least three months later compared with pre-vaccination infection [67]. A national cohort study of children and adolescents from the UK that included SARS-CoV-2-positive and -negative groups 6 months after a positive PCR test showed similar symptomatology among COVID vaccinated and unvaccinated participants, suggesting that the vaccination strategy against long COVID may not be effective in younger age groups [79]. Only one in five (20.3%) vaccinated individuals reported an improvement in symptoms after COVID-19 vaccination, while one in two (54.4%) of those living with long COVID did not notice any change after vaccination [75].

A comparative study in the UK showed that despite the reduction in the likelihood of long COVID with the Omicron variant family compared to the Delta variant, it is estimated that the number of people suffering from long COVID has actually increased due to the large number of Omicron infections [60]. A national cohort study of patients with long COVID from France showed that vaccination, even just with the first dose of a COVID vaccine, reduced the severity of symptoms and had a beneficial effect on all aspects of patients’ lives after 120 days compared with an unvaccinated control group [80]. A recent multicenter cross-sectional study concluded that previous SARS-CoV-2 infection caused by pre-Omicron variants was the strongest risk factor for long COVID and that vaccination in the pre-Omicron period was not associated with fewer long COVID symptoms [81].

### 6.3. The Potential Advantages of Vaccine-Induced vs. Hybrid Immunity in Long COVID

Evidence is accumulating to support vaccination, both pre- and post-SARS-CoV-2 infection, as a preventive strategy to reduce the risk of long COVID [82]. Nonetheless, more robust comparative observational studies, including randomized trials, are required to bring conclusive evidence on the vaccine effectiveness in preventing or ameliorating long COVID [63]. Further studies are thus warranted to enhance our understanding of long COVID and the impact of vaccination on its prevalence and course. So far, there is no evidence-based answer as to whether natural, vaccine-induced, or hybrid immunity offers better protection against long COVID.

Additional and appropriately designed studies are needed to address the impact of vaccination on hospitalization (vs. non-hospitalized), age (children and adolescents vs. adults), sex (females vs. males), and to clarify the role and impact of different types of vaccines and vaccine boosters on long COVID. Moreover, the impact of COVID-19 vaccination on long COVID during the dominance of different SARS-CoV-2 variants and the impact of reinfections on vaccinees with long COVID are also not clearly established [68,70]. Large vaccine trials to estimate rates of long COVID in vaccinees, as well as follow-up studies on vaccine efficacy in children by monitoring of breakthrough cases and prevalence of long COVID are required [63]. The effect of vaccines on different age groups and virus variants should also be better evaluated.

## 7. Long COVID in Children and Adolescents

### 7.1. Children May Also Suffer from Long COVID, but Less Frequently and Severely Than Adults

The clinical outcome of SARS-CoV-2 infections is affected to a large extent by age [83]. Infections are typically mild or asymptomatic in children and adolescents [84,85], and pediatric reinfections tend to be even milder than primary infections in most cases [86]. Severe illness, including a multisystem inflammatory syndrome (MIS-C), is an exception to this general rule [84,85]. Long COVID is a multisystemic condition seen in all ages. Thus, the long-term consequences of COVID-19 in the pediatric population can also manifest as long COVID [87,88]. Although there is no officially adopted and internationally recognized definition of long COVID in the pediatric population, most researchers agree that it entails a complex symptomatology that lasts for at least three months after the initial SARS-CoV-2 infection, it cannot be explained with alternative diagnosis, and it has a negative impact on everyday life [89].

A higher likelihood of long-lasting symptoms compatible with long COVID was found in children who had documented SARS-CoV-2 infection compared to the matched SARS-CoV-2 negative control group in most studies [2,90,91,92]. The ISARIC COVID-19 study, which included children and adolescents with documented SARS-CoV-2 infection and their household members, found that children can experience persistent multisystem symptoms months after mild COVID-19, although less frequently and less severely than adults [93]. The fact that long COVID is less commonly observed in children and adolescents than in adults may be related to the lower frequency of documented SARS-CoV-2 infection and the lower burden of COVID-19 in these age groups [90]. Other determinants, such as the typically lower prevalence of chronic diseases in younger compared to older age groups, may also be contributing factors. A large number of pediatric cases (up to 90%) are missed due to the lower probability of obtaining a positive PCR test result compared to adults despite seroconversion, suggesting a possible overestimation of the obtained long COVID prevalence. Compared with adults, children are less likely to seroconvert after primary infection, but more likely to have a waning response following COVID-19 [2]. Nevertheless, long COVID is not exclusively associated with symptomatic COVID-19, but it has also been described in children with asymptomatic SARS-CoV-2 infection [88].

### 7.2. Clinical Manifestations of Long COVID in Children and Adolescents

In general, post-hospitalization follow up of pediatric case studies showed higher estimated prevalence of long COVID than community-based studies [94]. There were differences in the prevalence (4–66%) and duration of long COVID symptoms as well as in the impact on the daily life of children [90]. In a recent study that evaluated long-term outcomes in 12,424 pediatric COVID survivors, the pooled prevalence of long-term symptoms was 23.36% and declined over time to ~15% at one year [95]. A survey among British school children showed that they often reported different symptoms regardless of their SARS-CoV-2 test results; however, some symptoms, for instance loss of taste and/or smell, were more common in those with a previous infection [96].

Similar to adults, children diagnosed with long COVID may have prolonged and debilitating symptoms, such as fatigue, headache, weakness, chest pain, dyspnea, anosmia/ageusia, persistent cough, muscle pain, loss of appetite, and weight loss [2,90,97]. Chronic fatigue is one of the most common symptoms of long COVID [90,91,97] present in up to 87% of children and adolescents with long COVID [90]. Considering this symptom given the serious public health issue of childhood obesity that may have been made worse by the COVID-19 pandemic, it is not surprising that children with obesity are likely at risk of developing severe disease and complications, in addition to type 2 diabetes and risk factors for heart disease, such as high blood pressure and high cholesterol, muscle and joint problems, and fatty liver disease. An increased risk of myocarditis, venous thromboembolic events, long-term pulmonary disfunction, renal failure, and type 1 diabetes in children who experienced COVID-19 was also observed [2,90]. Moreover, cognitive dysfunction [98], but also mental health problems that included depression, anxiety, sleep disorders, memory loss, stress-related disorders, mood swings, attention deficit, hyperactivity disorder, and even suicidal behavior, have been reported in children and adolescents with long COVID [90,93,99].

In a recent Israeli study, children with long COVID had more memory difficulties compared to a control group of uninfected children [100]. Together with previously identified risk factors for long COVID, such as female gender, adolescence, and presence of chronic conditions [98,101], and especially asthma [102], hospitalization ≥2 days, ≥4 reported symptoms, and age ≥14 years at first visit to the emergency department were significantly associated with the diagnosis of long COVID in children 90 days after the first visit [91]. Moreover, long COVID is primarily seen in older school-aged children [97,102,103]. Specifically, female adolescents have been found to be at particular risk of long COVID [104]. The pathological mechanism for the sex-related predisposition to long COVID remains unknown [105]. Older age, comorbidities, and symptomatic infection were shown to be risk factors for long COVID in children [106].

COVID-19 restriction measures had negative indirect effects on children’s fitness, mental health, and other health parameters. An additional problem is that neuropsychiatric symptoms can be part of long COVID but also a consequence of quarantine and social restrictions imposed during the pandemic [107]. Although such health parameters have improved significantly since the lifting of mitigation measures, pre-COVID levels have not been reached [108]. Despite the high variability in duration, clinical manifestations of long COVID in children usually resolve within five [99] or six months [93,109] at most.

### 7.3. Recommendations to Mitigate the Effects of Pediatric Long COVID

The importance of unraveling the puzzle of long COVID and the impact of persistent symptoms on the physical and psychological well-being of children and youth in the future is highlighted in most studies [2,90]. An appropriate follow-up of children after SARS-CoV-2 infection is required, particularly among those with severe infection and pre-existing comorbidities [91]. Institutional care services need to be made more accessible to respond to the growing burden of mental health epidemics accompanying the COVID-19 pandemic, including tailored psychosocial care services to affected children and adolescents in a variety of settings [98]. Despite the heterogeneity of research methodology and the lack of a standardized case definition, appropriate control groups, misclassification, and loss to follow-up, evidence of the devastating impact of long COVID on children’s health has increased significantly over time [89].

Future longitudinal and intervention studies, with proper SARS-CoV-2 negative control groups and consistent standardized case definitions [98], are necessary to understand the true prevalence of long COVID in children in order to define effective strategies in the future [2,93,99,101]. The selection of a control group presents an increasing challenge as the pandemic progresses, due to loss of data, asymptomatic course of the disease, or false negative tests [89]. Continuous evidence-based clinical guideline updates are needed to facilitate the diagnosis and treatment of pediatric long COVID [90,91,101]. Studies evaluating the long-term effects of COVID compared to the consequences of other viral infections, such as CMV, EBV, or enteric viral infections, are needed [109,110]. Multi-disciplinary long COVID clinics for children and adolescents could provide the greatest therapeutic opportunities in the future [92]. In the end, long COVID in children and youth is still a vaguely defined entity that urgently requires standardized definitions, as well as the core outcome set. Until these requirements are met, it is difficult to determine the real prevalence of this disorder and the burden in the pediatric population [110].

## 8. Conclusions

Based on conservative estimates, 65 million people currently suffer from long COVID and the number remains high in the post-Omicron phase of the pandemic [2]. Long COVID is a multisystem disease that can have devastating effects on all organ systems and may cause lifelong consequences. The risk of developing long COVID exists in both asymptomatic and symptomatic SARS-CoV-2 infection. Older age, female sex, high body mass index (BMI), smoking, diabetes, and having had a severe episode of COVID-19 are currently considered as risk factors for long COVID. Reinfections with SARS-CoV-2 also increase the likelihood of long COVID, particularly in women. Further studies are needed to decipher the pathological mechanism of sexual dimorphism in long COVID. Despite the ambiguities over the definition of this complex condition and the cause(s) of its heterogenous symptoms, answers on the prevention, treatment options, and rates of recovery from long COVID are gradually beginning to emerge [2,111]. It appears that the chance of recovery is higher during the first year, after which long COVID essentially becomes a chronic condition. Children and adolescents are not spared. Table 2 summarizes the key findings on the associations of long COVID with reinfection, vaccination, and affected population groups.

### Prevention and Treatment Strategies of Long COVID

For now, prevention of long COVID is based primarily on avoiding SARS-CoV-2 infection and reinfection, principally by adopting a number of non-pharmaceutical measures, such as wearing well-fitting masks, social distancing, air ventilation of indoor spaces, and hand hygiene. Vaccination also seems to reduce the risk of long COVID. Currently, available diagnostic and therapeutic options for long COVID are largely insufficient. Medications, including Nirmatrelvir during the acute phase of COVID-19 [112] and Metformin [113], are being evaluated and show promise for their ability to reduce the risk of adverse health outcomes associated with long COVID. The use of a drug repurposing strategy should be expanded to discover additional anti-COVID-19 drugs, especially since the development of resistance should be anticipated [114]. Furthermore, the role of antiviral medicinal plants should be further explored as adjunct or supportive therapy, both for the prevention of severe disease after reinfection and as anti-long COVID therapeutics. Machine learning and other artificial intelligence (AI) tools, such as deep learning (DL), and neural networks (NN), could aid in epidemic forecasting and identifying additional treatments for COVID-19 and long COVID [115]. In this respect, specific models have been developed to represent and eventually estimate the risk of long COVID occurrence, when considering the various risk factors [116]. Such models that may be retrained and tuned based on the needs of individual studies, also achieve the urgent goal of identifying potential long COVID in patients for clinical trials [116]. The continuation of basic research will allow for a better understanding of the pathophysiology and mechanisms of long COVID, with the goal to accelerate clinical trials to rigorously test new treatments for long COVID.

## Figures and Tables

**Table 1:** Host and viral risk factors potentially associated with increased likelihood of long COVID after SARS-CoV-2 reinfection.

	Factor Type	Risk Factor for Long COVID upon Reinfection
**Host**	Biological	Female sex
		Older age ^1^
		Certain comorbidities (e.g., type 2 diabetes)
		Having had severe COVID-19 (particularly during the first few weeks of illness) ^2^
	Lifestyle	Obesity
		Smoking
**Viral**	SARS-CoV-2 variant	Omicron ^3^

^1^ Older age seems to hold as a risk factor for long COVID after SARS-CoV-2 reinfection also in children (probably >2 years) and adolescents. ^2^ With a gradient/descending order of severity and likelihood of long COVID after SARS-CoV-2 reinfection as follows: Intensive Care Unit (ICU) vs. hospitalization vs. Symptomatic vs. Mild vs. Asymptomatic infection. ^3^ Reflecting the increased number of SARS-CoV-2 reinfections with subvariants of the Omicron family.

**Table 2:** Summary of key findings on the associations of long COVID with reinfection, vaccination, and affected population.

	Key Findings Regarding Long COVID	Main Reference(s)
**Reinfection**	Reinfections increase the likelihood of long COVID (and additional complications, e.g., cardiac, pulmonary, neurological, in older subjects).	[55]
	The risk of developing long COVID symptoms is significantly lower after asymptomatic (compared to symptomatic) reinfection.	[56]
	Long COVID cases have been increasing upon reinfection with Omicron subvariants.	[57,58,60]
**Vaccination**	Vaccination against (severe) COVID-19 seems to also protect from long COVID (reduced risk by 15–41%).	[63,66,67]
	Two vaccine doses (of the primary scheme) are more effective than one dose.	[64,68,69,70]
	No difference in relation to the type of received vaccine.	[70]
**Affected population**	Children may also suffer from long COVID, but less frequently and less severely than adults.	[87,88,93]
	Chronic fatigue is one of the most common symptoms of long COVID present in up to 87% of children and adolescents with long COVID.	[90,91,97]
	Older age, comorbidities, and symptomatic infection are risk factors for long COVID in children.	[106]

## Data Availability

Not applicable.
